# *OsPG1* Encodes a Polygalacturonase that Determines Cell Wall Architecture and Affects Resistance to Bacterial Blight Pathogen in Rice

**DOI:** 10.1186/s12284-021-00478-9

**Published:** 2021-04-21

**Authors:** Yongrun Cao, Yue Zhang, Yuyu Chen, Ning Yu, Shah Liaqat, Weixun Wu, Daibo Chen, Shihua Cheng, Xinghua Wei, Liyong Cao, Yingxin Zhang, Qunen Liu

**Affiliations:** 1 State Key Laboratory of Rice Biology, China National Rice Research Institute, Zhejiang, 310006 Hangzhou China; 2 Key Laboratory for Zhejiang Super Rice Research, China National Rice Research Institute, Zhejiang, 310006 Hangzhou China

**Keywords:** Rice, Leaf tip necrosis, Polygalacturonase, Bacterial blight, Cell wall

## Abstract

**Background:**

Plant cell walls are the main physical barrier encountered by pathogens colonizing plant tissues. Alteration of cell wall integrity (CWI) can activate specific defenses by impairing proteins involved in cell wall biosynthesis, degradation and remodeling, or cell wall damage due to biotic or abiotic stress. Polygalacturonase (PG) depolymerize pectin by hydrolysis, thereby altering pectin composition and structures and activating cell wall defense. Although many studies of CWI have been reported, the mechanism of how PGs regulate cell wall immune response is not well understood.

**Results:**

Necrosis appeared in leaf tips at the tillering stage, finally resulting in 3–5 cm of dark brown necrotic tissue. *ltn-212* showed obvious cell death and accumulation of H_2_O_2_ in leaf tips. The defense responses were activated in *ltn-212* to resist bacterial blight pathogen of rice. Map based cloning revealed that a single base substitution (G-A) in the first intron caused incorrect splicing of *OsPG1*, resulting in a necrotic phenotype. *OsPG1* is constitutively expressed in all organs, and the wild-type phenotype was restored in complementation individuals and knockout of wild-type lines resulted in necrosis as in *ltn-212*. Transmission electron microscopy showed that thicknesses of cell walls were significantly reduced and cell size and shape were significantly diminished in *ltn-212*.

**Conclusion:**

These results demonstrate that *OsPG1* encodes a PG in response to the leaf tip necrosis phenotype of *ltn-212.* Loss-of-function mutation of *ltn-212* destroyed CWI, resulting in spontaneous cell death and an auto-activated defense response including reactive oxygen species (ROS) burst and pathogenesis-related (PR) gene expression, as well as enhanced resistance to *Xanthomonas oryzae* pv*. oryzae* (*Xoo*). These findings promote our understanding of the CWI mediated defense response.

**Supplementary Information:**

The online version contains supplementary material available at 10.1186/s12284-021-00478-9.

## Introduction

Rice (*Oryza sativa* L.) is not only the world’s most important food crop for more than half of the world’s population, but also a model species for genetic, cytological, and agricultural studies (Itoh et al. 2005). As the main photosynthetic organs, the development status of rice leaves is closely related to the final yield. Premature withering of rice leaves has a significant impact on plant growth and yield. Thus, understanding the molecular mechanisms of leaf development and identifying genes causing premature withering are of great interest to both plant biologists and plant breeders.

Leaf necrosis starts to appear mainly at the apical meristem. The necrotic tips may be a specific regulation of the plant response to changes in the external environment or gene expression in vivo. The factors that induce leaf tip necrosis have been described at a molecular level in recent studies. The ectopic content of elements may result in leaf tip necrosis. The rice *leaf tip necrosis 1* (*ltn 1*) mutant results in excessive accumulation of phosphate in shoots and thus causes plants to develop a leaf tip necrotic phenotype (Hu et al. 2011). *OsPT8* is involved in phosphate homeostasis in rice, and overexpressing *OsPT8* resulted in excessive phosphate in shoots and chlorosis and necrosis in leaf tips under high phosphate (0.3 mM) supply (Jia et al. 2011).

Leaf tip necrosis or withering usually occur at the beginning of leaf senescence. A rice mutant *Leaf Tip Senescence 1* (*LTS1*) resulted in dwarfism and withered leaf tips, ultimately causing early leaf senescence (Wu et al. 2016). The loss of function of *DEL1* resulted in the leaf apex and leaf margin exhibiting a faint yellow color after germinating for 5 days, while leaf tips exhibited withering and cracking at the maturity stage (Leng et al. 2017). The phenotype of necrotic leaf tips correlates with plant resistance. *Lr34* confers durable and partial field resistance to wheat against an obligate biotroph associated with leaf tip necrosis (Singh. 1992). Transforming resistant *Lr34* into Nipponbare allele showed increased resistance against multiple isolates of *Magnaporthe oryzae* and plants also developed a typical, senescence-based leaf tip necrosis phenotype (Krattinger et al. 2016). The same phenotype also occurred in transgenic barley with *Lr34* (Risk et al. 2013). Necrotic leaves caused by programmed cell death (PCD) also alter cell wall structure. Mutation of a *PECTATE LYASE-LIKE* gene. *DEL1*, led to PCD and changes in cell wall composition and structure in rice (Leng et al. 2017). In other cases, the cell wall is degraded in abscission zones and is a key event in lace plant PCD (Gunawardena et al. 2007).

The typical plant cell wall consists of a primary cell wall, secondary cell wall and middle lamella, comprised of various carbohydrate-based polymers (cellulose, pectins and hemicelluloses) (Sarkar et al. 2009). PGs belong to one of the largest hydrolase families (GH28) and are expressed in a wide range of tissues and developmental stages in plants and fungus (Markovic and Janecek. 2001). PGs are one of the primary cell wall hydrolases affecting cell wall architecture by degrading pectin (Micheli. 2001). PGs have been identified to have multiple functions in a diverse range of processes including fruit ripening, organ abscission, pollen maturation, pathogen infection and plant resistance to pathogens (Atkinson et al. 2002; Wang et al. 2005; Villarreal et al.; 2009). For example, MdPG1 and MdPG2 regulate apple fruit softening and are induced by ethylene (Wakasa et al. 2006; Li et al. 2010). *FaPG1* was up-regulated during strawberry fruit ripening and silencing *FaPG1* by antisense transformation significantly reduced strawberry fruit softening (Quesada et al. 2009). Down-regulation of *PpBGAL10* and *PpBGAL16* expression inhibited cell wall degradation and ethylene production and delayed peach fruit softening by decreasing PG and pectin methyl esterases (PME) activity (Liu et al. 2018).

In *Arabidopsis*, PGs mainly function in growth and developmental processes including cell separation and expansion. *ARABIDOPSIS DEHISCENCE ZONE POLYGALACTURONASE 1* (*ADPG1*) and *ADPG2* are essential for silique dehiscence; *ADPG2* and *QUARTET2* (*QRT2*) contribute to floral organ abscission; and all three genes contribute to anther dehiscence (Ogawa et al. 2009). Mutation of *QRT3* resulted in failure of microspore separation during pollen development (Rhee et al. 2003). *POLYGALACTURONASE INVOLVED IN EXPANSION1* (*PGX1*) and *PGX2* function in cell expansion in seedling growth and rosette expansion in adult plants; overexpressing them resulted in enhanced hypocotyl elongation (Xiao et al. 2014; Xiao et al., 2017). Different from the two previously characterized *PGX* genes, *PGX3* functions in seed germination, which contributes to the determination of etiolated hypocotyl length and root length in young seedlings (Rui et al. 2017). Microbial PGs stimulate plant cell walls to release oligogalacturonides (OGs) to activate defense responses during infections. Phytopathogenic fungi secrete PGs to degrade the pectic homogalacturonan (HG) backbone and aid host colonization by degrading CWI (Annis and Goodwin. 1997). Transgenic expression of a fungal PG in tobacco and *Arabidopsis* results in higher resistance to microbial pathogens and constitutively activated defense responses (Ferrari et al. 2007).

Although many PGs have been genetically and biochemically characterized in plants, few studies investigating PGs in rice have been reported. *OsBURP16* belongs to the PG1*β*-like subfamily of BURP-family genes and encodes one putative PG1*β* subunit precursor, which showed enhanced PG activity and reduced pectin content and increased abiotic stress sensitivity in rice when it was overexpressed (Liu et al. 2014). In this study, we report the isolation of a leaf tip necrosis mutant named *ltn-212* that exhibits necrotic leaf tips. The mutation in *ltn-212* led to H_2_O_2_ accumulation and cell death in the necrotic part, but also caused enhanced resistance to bacterial blight pathogen *Xoo*. Using a map-based cloning strategy, we isolated the gene that encodes OsPG1. Our data suggested that *OsPG1* is responsible for leaf tip necrosis in *ltn-212* and functions directly in defense responses by mediating cell wall structure.

## Results

### Phenotypic Characteristics of *ltn-212*

**Fig. 1:**
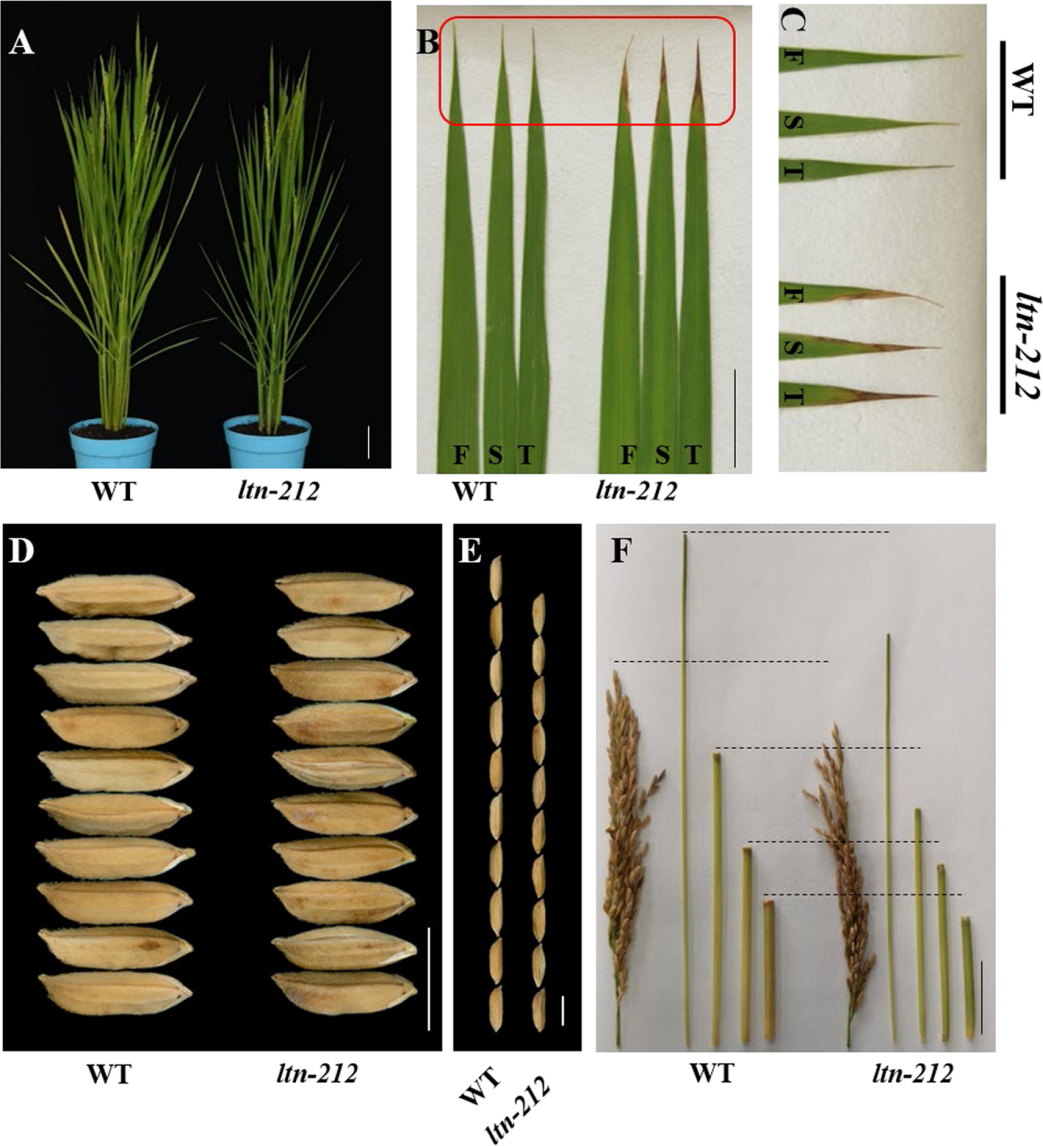
Leaf tip necrosis and agronomic traits of *ltn-212*. **a** Phenotype of WT and *ltn-212* at heading stage (scale bar = 10 cm). **b** Leaf tip necrosis identification of WT and *ltn-212* at heading stage (scale bar = 5 cm). **c** Enlarge of red part of Fig. **b. d-e** The phenotype of grain width and grain length (scale bar = 10 mm). **f** Comparison of panicles and culm length of WT and *ltn-212* (scale bar = 5 cm).** indicates significance at *P* ≤ 0.01 and * indicates significance at *P* ≤ 0.05 (Student’s *t* test). F: The first top leaf. S: The second top leaf. T: The third top leaf

We isolated a leaf tip necrosis mutant from an ethyl methane sulfonate (EMS)-derived mutant population in the JiaHe212 background, which we named *ltn-212* (*leaf tip necrosis*). Under natural summer field conditions, the leaf tip of *ltn-212* had a small necrosis region and dark brown necrotic spots were formed at the tillering stage (Additional file 1: Figure S1A-B). Necrosis or withering at the leaf tip is typically the beginning of plant senescence and extends to the bottom of the leaf as the plant grows. Unlike other plant materials, initial necrosis of *ltn-212* only occurred at all leaf tips at the tillering stage and developed into 3–5 cm of dark brown necrotic tissue until ripening stage (Fig. 1a-c). In general, the necrosis changed during plant growth but was limited to the leaf tip in *ltn-212*.

The leaf is the mainly photosynthetic organ for energy assimilation of rice. As a result, *ltn-212* significantly reduced plant height, 1000-grain weight, and grain length, compared to wild-type (WT, JiaHe212), but there were no changes in grain width or tiller number (Fig. 1d-e, Additional file 1: Figure S2A-D). The reduced plant height for *ltn-212* was due to shortened panicles along with shortened first three internodes compared with WT (Fig. 1f, Additional file 1: Figure S2E). The data suggested that the leaf-necrosis mutation also significantly affected agronomic performance.

### H_2_O_2_ Accumulation and Cell Death Occur in *ltn-212*

**Fig. 2:**
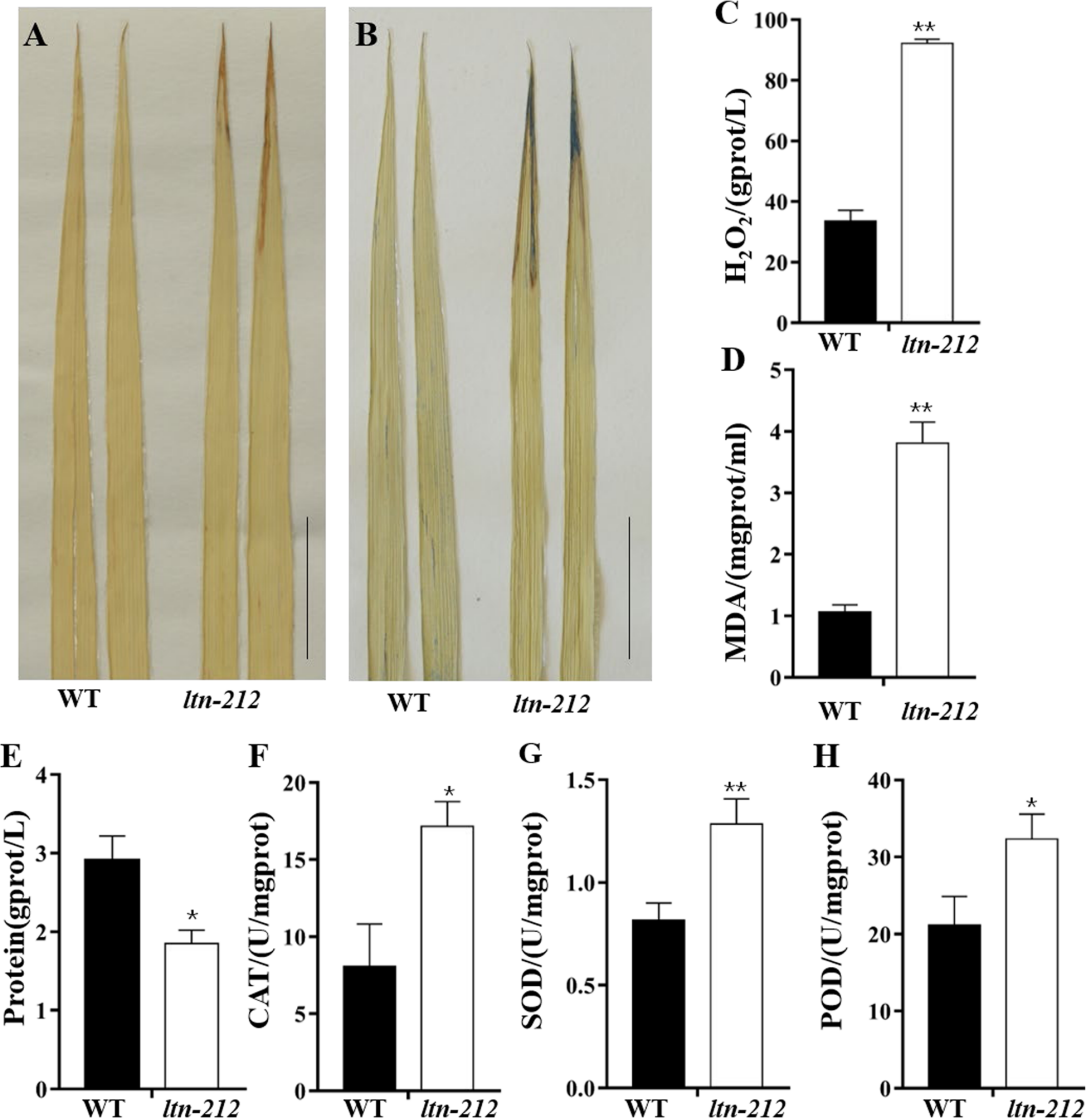
Histochemical staining of WT and *ltn-212*. **a-b** DAB staining and Evans blue staining at heading stage (scale bar = 5 cm). **c-d** H_2_O_2_ content and MDA content at heading stage (*n* = 3). **e-h** Total protein content and the activity of reactive oxygen species scavenging enzymes (CAT, SOD, POD, n = 3). Data are means ± SD. ** indicates significance at P ≤ 0.01 and * indicates significance at P ≤ 0.05 (Student’s *t* test)

Necrosis has been defined as a type of cell death, mainly caused by ROS burst and H_2_O_2_ accumulation (Van and Dat, 2006). To determine whether the development of necrosis in *ltn-212* involves altered H_2_O_2_ accumulation and cell death, we performed 3,3-Diaminobenzidine (DAB) and Evans blue staining. DAB staining results in the formation of reddish-brown formazan precipitates indicative of H_2_O_2_ accumulation. The red brown precipitate was observed on the *ltn-212* leaf tip, whereas no precipitate was observed in WT (Fig. 2a). In addition, the H_2_O_2_ contents in the *ltn-212* leaf tips was approximately 3-fold higher than in WT leaf tips (Fig. 2c). Evans blue staining is an indicator of irreversible membrane damage or cell death (Liu et al. 2008). The *ltn-212* leaf tip with necrosis had blue stained cells, whereas no staining was observed in *ltn-212* leaves without necrosis or WT (Fig. 2b). To confirm membrane damage and cell death, we further measured the levels of malonaldehyde (MDA). The *ltn-212* mutant showed significantly higher levels of MDA content compared to WT (Fig. 2d).

Plants can remove ROS by synthesizing anti-oxidative enzymes when injured and suffering from stress. We quantitatively determined the activity of these enzymes in *ltn-212* and WT leaves. The results indicated that content of total protein significantly decreased in *ltn-212* compared to WT. The activities of catalase (CAT) and superoxide dismutase (SOD) and peroxidase (POD) increased in *ltn-212* plants, by 17.15 *U/mgprot*, 1.28 *U/mgprot* and 32.35 *U/mgprot* in *ltn-212* leaf tips, respectively, which were approximately 2.1-, 1.6- and 1.5-fold higher than the levels of 8.08 *U/mgprot*, 0.82 *U/mgprot* and 21.19 *U/mgprot* in the WT, respectively (Fig. 2e-h). Taken together, these results demonstrate that the formation of leaf necrosis was associated with ROS accumulation and irreversible membrane damage in the *ltn-212* cells.

### Activation of Defense Responses in *ltn-212*

**Fig. 3:**
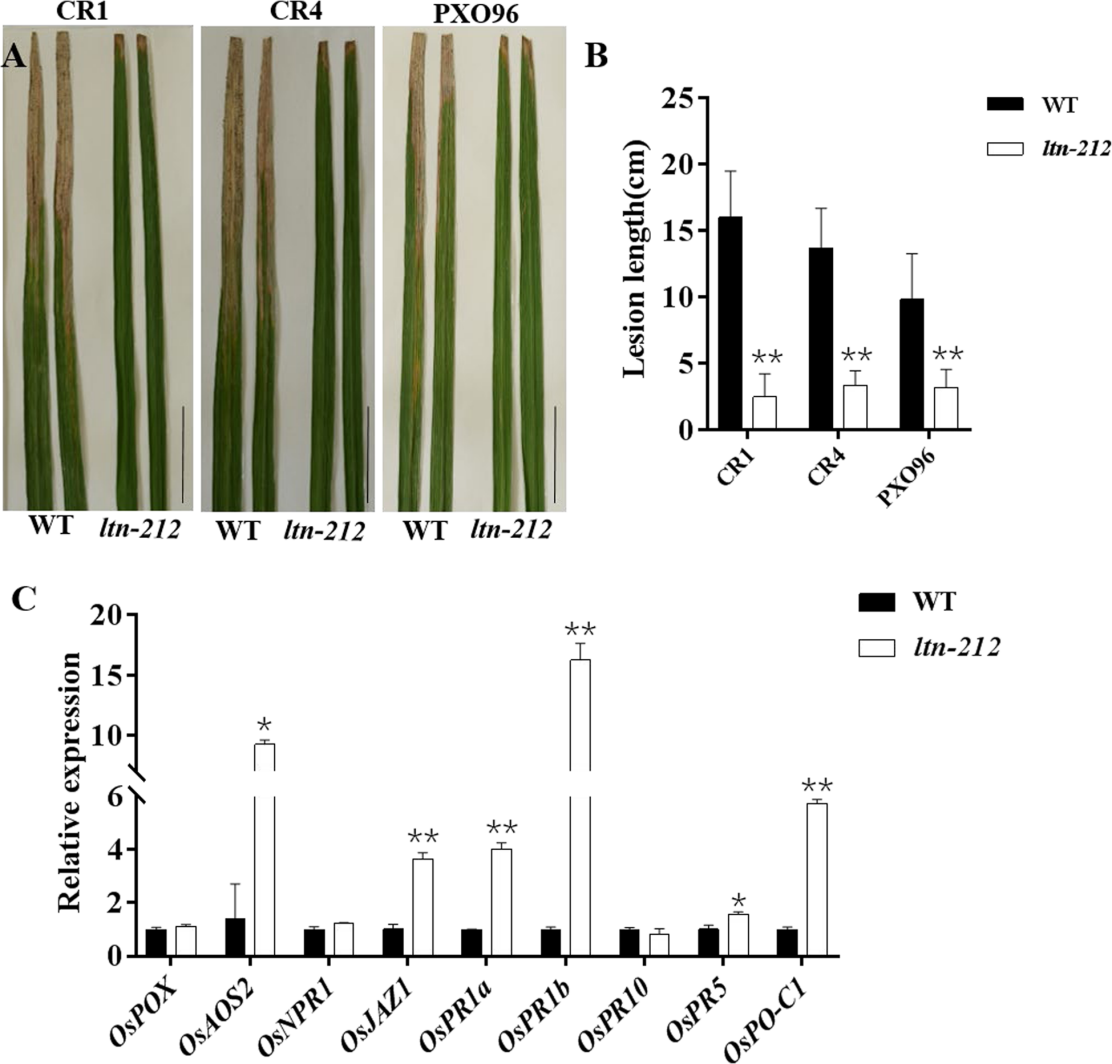
Enhanced resistance in *ltn-212* to *Xoo*. **a** Disease symptoms of WT and *ltn-212* leaves after *Xoo* inoculation at 15 days (scale bar = 5 cm). **b** Lesion lengths measured at 15 days after inoculation with 3 different *Xoo*. Data are means ±SD of at least 15 leaves. **c** Expression analysis of defense-related genes. Data are means ± SD. ** indicates significance at P ≤ 0.01 and * indicates significance at P ≤ 0.05 (Student’s *t* test)

Accumulation of ROS commonly activates defense responses that result in enhanced resistance to one or more pathogens. To examine whether the *ltn-212* mutant also gains disease resistance, we inoculated the mutant and the WT plants with isolates of three *Xoo* pathotypes virulent against the WT using the leaf clipping method at the tillering stage. The results showed that *ltn-212* exhibited significantly enhanced resistance to races CR1, CR4, and PXO96 compared to WT (Fig. 3a-b). To check whether the enhanced disease resistance response stemmed from elevated expression of defense related genes, we measured the expression of nine PR genes by real-time quantitative PCR analysis. These genes were mainly involved in SA and JA signaling pathways. The results showed the mRNA levels of *OsJAZ1*, *OsPR1a*, *OsPR1b*, *OsPR5* and *OsPO-C1* significantly increased compared to WT; in particular, the expression of *OsPR1b* was upregulated 15-fold in *ltn-212* compared to WT (Fig. 3c). Thus, we speculated that the leaf tip necrosis also triggered a defense response, resulting in enhanced resistance to *Xoo* in *ltn-212.*

### Map-Based Cloning of *OsPG1*

**Fig. 4:**
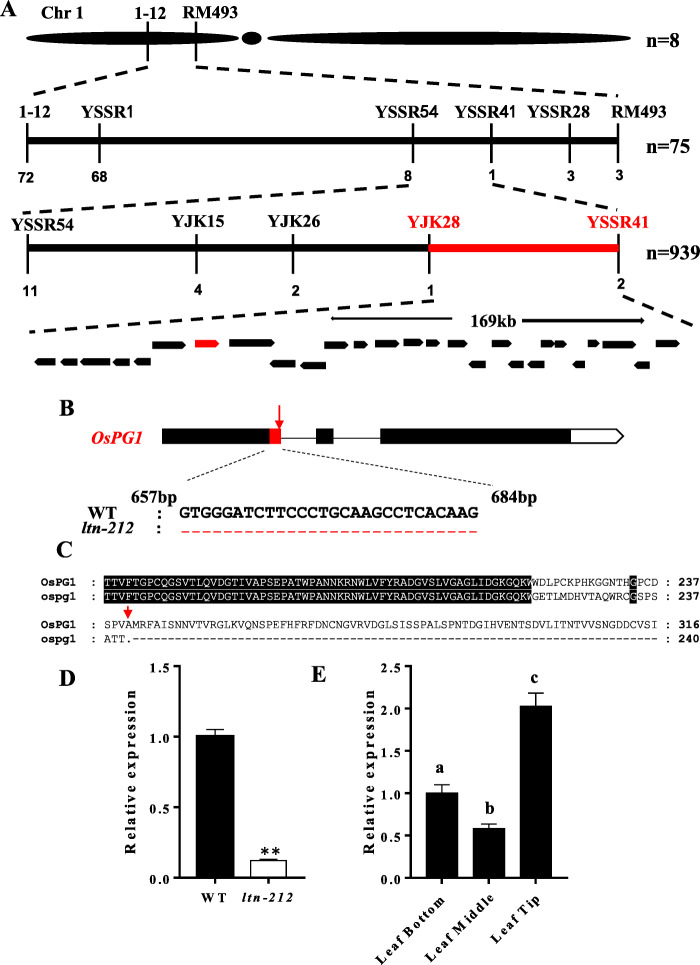
Cloning analysis of the *OsPG1*. **a** Fine mapping of *OsPG1*. **b** Gene structure and the mutation of genome and cDNA in *OsPG1*. The red arrow indicates the mutant site and the red dotted line indicates the incorrected splicing sequences. **c** Protein sequence alignment of OsPG1 and ospg1. **d** Expression analysis of *OsPG1* in WT and *ltn-212* leaves (n = 3). **e** Expression analysis of *OsPG1* in WT leaf bottom, middle and tip (n = 3). Data are means ± SD. ** indicates significance at P ≤ 0.01 and * indicates significance at *P* ≤ 0.05. **a, b, c** indicates significance at P ≤ 0.01 (Student’s *t* test)

To determine the genetic control of the mutation, we crossed *ltn-212* and the WT JiaHe212. F_1_ progenies had a normal phenotype matching that of WT and in the F_2_ segregating populations, normal and mutant phenotypes showed a typical segregation ratio of 3:1 (158:42, χ^2^ = 1.56), indicating that the leaf tip necrotic phenotype of *ltn-212* was controlled by a single recessive nuclear gene. To map the gene responsible for the *ltn-212* mutant phenotypes, an F_2_ mapping population was constructed by crossing *ltn-212* with the *indica* variety ZH8015. Eight F_2_ plants with the *ltn-212* phenotype were used for primary mapping, and the mutation was located on the short arm of chromosome 1 between markers 1–12 and RM493 (Fig. 4a). By using 75 homozygous mutant plants, the mutation was further located to markers YSSR54 and YSSR41 (Fig. 4a). Subsequently, using an additional 939 F_2_ mutant individuals and three newly developed polymorphic markers, the mutation was finally narrowed down to an approximately 169 Kb region between markers YJK28 and YSSR41 (Fig. 4a). Twenty-seven open reading frames (ORFs) were predicted in this region (Fig. 4a).

Sequencing and comparison of those ORFs cloned from *ltn-212* and WT revealed that the seventh ORF (*LOC_Os01g19170*) had a single base substitution (G-A) in the first intron (Fig. 4b, Additional file 1 Figure S3). We then sequenced cDNA of *LOC_Os01g19170* from both genotypes. The sequence alignment revealed that the mutation disrupted the recognition site for an exon, causing incorrect splicing 28 bp at the first exon in *ltn-212* (Fig. 4b, Additional file 1: Figure S3). This splicing error was predicted to introduce a premature stop codon at the 240th amino acid (Fig. 4c). We performed qRT-PCR to understand if the mutation caused changes in expression of *OsPG1* and the expression level in leaves at different positions. The expression of *OsPG1* was significantly down-regulated in *ltn-212* leaves compared to WT leaves and the mRNA level was highest in leaf tips (Fig. 4d-e). On the basis of these data, we speculated that *OsPG1* was responsible for the necrotic leaf tips.

### Functional Analysis and Verification of *OsPG1*

**Fig. 5:**
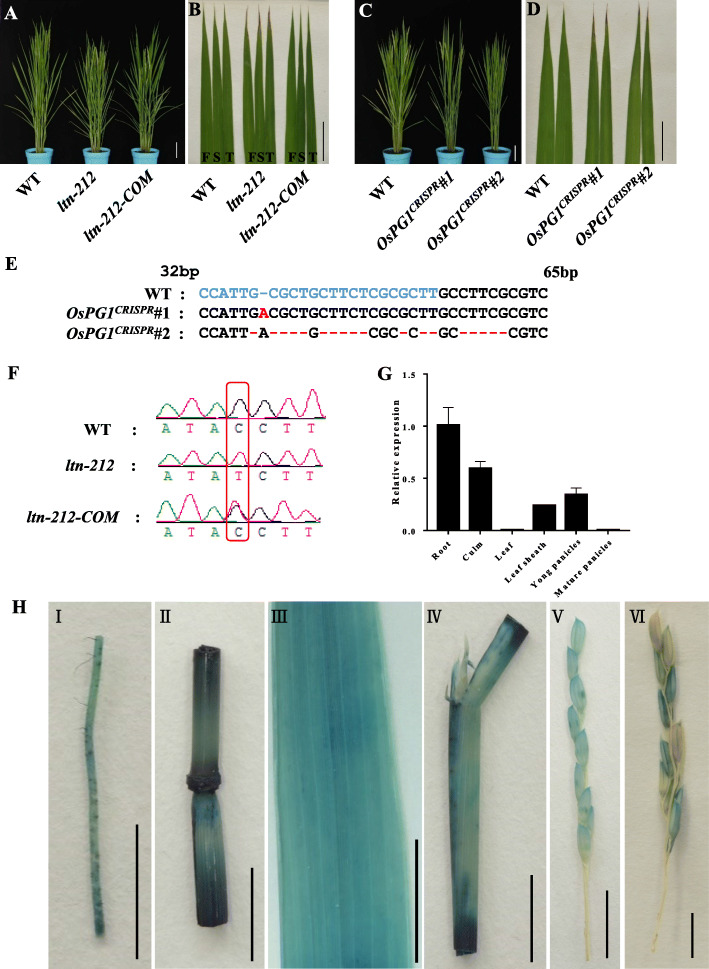
Genetic complementation, knock-out and expression analysis of *OsPG1*. **a** Phenotype of WT, *ltn-212* and T_1_ complementation plant (scale bar = 10 cm). **b** Leaf tip necrosis disappear on the leaves of T_1_ complementation plant (scale bar = 5 cm). **c** Phenotype of WT, and *OsPG1* knock-out individuals (*OsPG1*^*CRISPR*^*#1*, *OsPG1*^*CRISPR*^*#2*) at JiaHe212 background (scale bar = 10 cm). **d** Leaf tip necrosis on the leaves of knock-out individuals (*OsPG1*^*CRISPR*^*#1*, *OsPG1*^*CRISPR*^*#2*) (scale bar = 5 cm). **e** Mutational sites of knock-out individuals (*OsPG1*^*CRISPR*^*#1*, *OsPG1*^*CRISPR*^*#2*). The blue color words indicate target sequence and the red color words indicate insertion and deletion mutations. **f** The T_1_ transgenic plants were verified by sequencing of the mutation site. **g** Expression levels of *OsPG1* in various tissues (n = 3). **h** Gus staining of OsPG1 promoter–GUS reporter transgenic plants (scale bar = 1 cm). I: Roots. II:Colum. III: leaf. IV: Sheath. V: Young spikelet. VI: Mature spikelet. Data are means ± SD. ** indicates significance at P ≤ 0.01 and * indicates significance at P ≤ 0.05 (Student’s *t* test)

To verify whether the incorrect splicing of *OsPG1* was responsible for the *ltn-212* phenotype, we constructed a complementation vector containing a WT-derived 5790 bp genomic DNA fragment consisting of the entire *OsPG1* coding region, 2826 bp upstream and 1098 bp downstream sequences, and introduced it into *ltn-212* using *Agrobacterium-*mediated transformation. The resulting complemented plants are referred to as *ltn-212-COM*. No necrosis appeared on the leaf tip of any *ltn-212-COM* plants at any point in the life cycle (Fig. 5a-b). We carried out genotyping of T_1_ plants with no necrosis to check for the transgene by sequencing the region of *OsPG1* that contains the mutation. We found that all progenies were heterozygous at the mutation site (Fig. 5e). Furthermore, we transformed a CRISPR-Cas9 construct targeting the first exon of *OsPG1* into the WT, and obtained *OsPG1* knock-out individuals (Fig. 5c-d). All the knockout plants exhibited the leaf tip necrotic phenotype (Fig. 5c-d, e). Together, our results indicate the mutation of *OsPG1* was responsible for the formation of the necrotic leaf tip.

Sequence comparison between genomic DNA and cDNA showed that *OsPG1* is composed of three exons separated by two introns. The coding sequence (CDS) of *OsPG1* consists of 1512 nucleotides, and encodes a putative PG with a 504 amino acid protein. PG is one of the hydrolases of pectin and is involved in controlling the stability of cell wall structure. The typical PG genes in plants and fungi have four conserved domains: Domain I (SPNTDG), Domain II (GDDC), Domain III (CGPGHGISIGSLG), and Domain IV (RIK). Sequence alignment of PGs homologous among rice, *Arabidopsis thaliana*, *Brassica rapa* L. ssp. *pekinensis*, *Ciboria shiraiana*, *Rhizoctonia solani*, and *Glycine max* revealed that OsPG1 was highly conserved (Additional file 1: Figure. S4). The rice genome contains 45 PG-like genes. Phylogenetic analysis of the PG in rice showed that OsPG1 was classified into Clade A. Genes in Clade A are mainly expressed at fruiting and abscission (Additional file 1 Figure S5). The results indicate that *OsPG1* is a typical PG gene.

### Expression Pattern of *OsPG1*

To elucidate the spatial and developmental expression pattern of *OsPG1* in rice, qRT-PCR assays were performed in roots, culm, leaves, sheath, young panicles, mature panicles in the WT. *OsPG1* was highly expressed in the roots, culm, sheath and young panicles while expression was barely detectable in leaves and mature panicles (Fig. 5g). We generated transgenic plants of *OsPG1pro*::GUS expressing the *β*-glucuronidase (GUS) reporter gene driven by the native promoter of *OsPG1* to more precisely examine the spatial and temporal expression patterns of *OsPG1*. GUS staining showed activity of the *OsPG1* promoter in all tissues examined (Fig. 5h). In summary, the results showed that *OsPG1* was broadly expressed in all tissues.

### Mutation of *OsPG1* Altered Cell Wall Structure and Composition and Enhanced Resistance to Bacterial Blight Pathogen

**Fig. 6:**
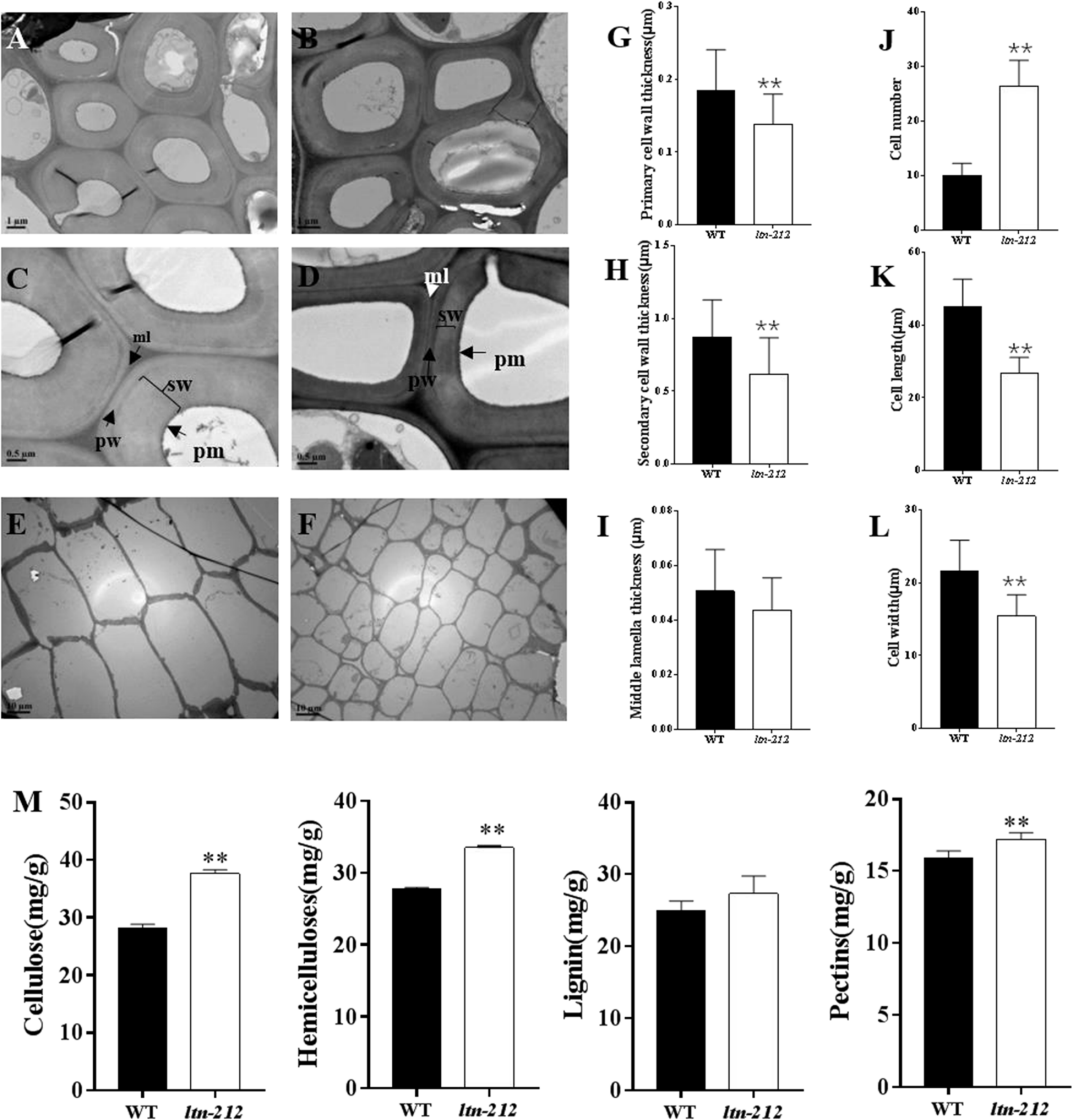
Cell wall structure in WT and *ltn-212*. **a-b** TEM micrographs of the bundle sheath fiber cells in leaves of WT (**a**) and *ltn-212* (**b**) (scale bar =1 μm). **c** Magnification in (**a**), and D. magnification in (**b**) (scale bar = 0.5 μm). E-F. TEM micrographs of the internode cell of WT (**e**) and *ltn-212* (**f**) (scale bar = 10 μm). **g-i** Statistical analysis of the primary cell wall, secondary cell wall, and middle lamella thicknesses of bundle sheath fiber cells in leaves of WT and *ltn-212*. Data are means ± SD of 20 cells. **j-l** Statistical analysis of the cell number, cell length and cell width of WT and *ltn-212*. Data are means ±SD of 30 cells. **m** The cell wall composition of WT and *ltn-212* (*n* = 5). ** indicates significance at P ≤ 0.01 and * indicates significance at P ≤ 0.05 (Student’s *t* test). ml: middle lamella, pw: primary cell wall, sw: secondary cell wall, pm: plasma membrane

Previous studies have demonstrated that pectin is fundamental and the most complex polysaccharide in plant cell walls (Bonnin et al., 2014). Functional analysis of *OsPG1* suggested that the cell wall structure in mutant plants may be altered. We therefore analyzed the structure of *ltn-212* and WT plants using Transmission Electron Microscope (TEM). The results revealed that the cell wall thicknesses of bundle sheath fiber cells in *ltn-212* were altered (Fig. 6a-d.). The thicknesses of the primary and secondary cell walls were reduced by 25.2% and 29.2% compared to WT, respectively, but there were no significant differences of middle lamella in *ltn-212* and WT (Fig. 6g-i). The cell wall thickness in different leaf sites of *ltn-212* were reduced compared to WT (Additional file 1: Figure. S6). Moreover, the results of TEM in internodes showed that the cell numbers in *ltn-212* increased by approximately 2.6-fold compared to the levels in WT; the cell length and width were reduced by 40.6% and 39.6%, respectively (Fig. 6e-f, j-l). To examine whether *ospg1* affected cell wall composition, we analyzed the cellulose, hemicellulose, lignin and pectin contents of WT and *ltn-212*. The cellulose, hemicellulose and pectin contents of *ltn-212* were increased by 33.6%, 20.8%, and 8.1%, respectively, while the lignin was no significantly changed (Fig. 6m). The results indicate *OsPG1* mutation casues complex cell wall structure and composition altertion.

**Fig. 7:**
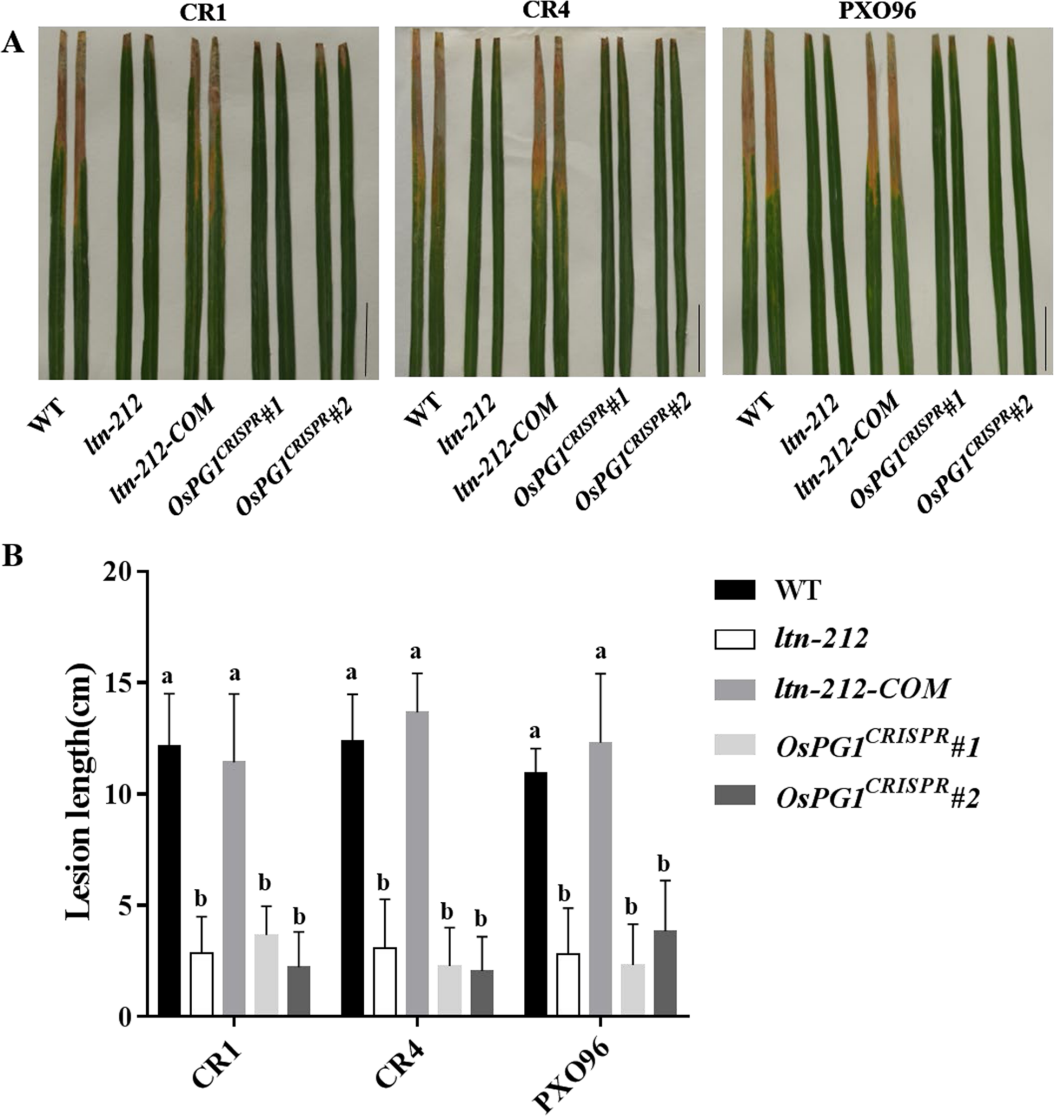
Disease reactions of the WT, *ltn-212* and transgene plants to *Xoo*. A. Disease symptoms of WT, *ltn-212*, *ltn-212-COM, OsPG1*^*CRISPR*^*#1*, and *OsPG1*^*CRISPR*^*#2* leaves after *Xoo* inoculation at 15 days (scale bar = 5 cm). B. Lesion lengths measured at 15 days after inoculation with 3 different *Xoo* strains. Data are means ±SD of at least 15 leaves. a, b indicates significance at P ≤ 0.01 (Student’s *t* test)

The cell wall is the first barrier against pathogen infection. To determine whether changes in cell wall structure enhanced resistance to the bacterial blight pathogen, we performed a disease resistance assessment with CR1, CR4, and PX096 of *ltn-212-COM* and *OsPG1*^*CRISPR*^ plants. We observed that, similar to WT, the *ltn-212-COM* plants were more susceptible than *ltn-212* plants to the compatible *Xoo*, while *OsPG1*^*CRISPR*^ exhibited shorter lesions similar to those in *ltn-212*, compared to WT (Fig. 7a-b). These results suggest that the *OsPG1* mutation causes structural alterations in cell walls, and *OsPG1* negatively regulates disease resistance in rice.

## Discussion

It has been more than 50 years since the PG gene was first identified, but few PG genes have been reported in crops. In the present study, we identified a novel leaf tip necrotic mutant *ltn-212* in an EMS mutagenesis mutant population of rice cultivar JiaHe212. *OsPG1* was identified as LOC_Os01g19170, which encodes a PG. Functional complementation and a knock out assay confirmed that the necrotic leaf tips were caused by incorrect splicing of *OsPG1*, indicating that *OsPG1* functions directly in regulating normal leaf growth and development.

The necrosis or withering of leaves commonly accompanies PCD (Hong et al., 2018; Ruan et al., 2019). *ZmPGH1*, a PG gene homolog, functions as a suppressor of PCD in maize (He et al. 2019). *ltn-212* showed dark brown necrotic spots in leaf tips at the tillering stage, which developed into 3–5 cm of dark brown necrosis. Evans blue staining and increased MDA content of *ltn-212* showed obvious cell death in leaf tips. TEM observation of the *ltn-212* cells showed that areas without necrosis did not have cellular structure destruction or chloroplast degradation (Data not provide). Thus, we speculated that the necrotic leaf tips of *ltn-212* were due to cell death and *OsPG1* is a general suppressor of PCD in rice. The production of intracellular ROS is closely associated with cell death (Zurbriggen et al. 2010). As expected, DAB staining and H_2_O_2_ content in leaf tips occurred at higher levels in the *ltn-212* mutants than in WT plants. To eliminate the accumulation of oxidative damage caused by ROS, plants have evolved ROS scavenging enzymes, like SOD, POD, and CAT. In the present study, the activities of three scavenging enzymes were significantly increased, which indicated that the balance between ROS production and scavenging was uncontrolled. ROS burst usually causes serious damage to plants, including brown spots, wilting and necrosis on leaves (Ma et al. 2019; Sathe et al. 2019; Rani et al., 2020). In *ltn-212*, the necrosis caused by uncontrolled ROS was confined to leaf tips. Interestingly, the qPCR results of *OsPG1* in leaf showed that tips had the highest expression levels. This indicates that leaf tips have the highest mRNA levels, which might be responsible for tip necrosis.

PGs are essential in plant growth and development. In *Arabidopsis*, *PGX1*, *PGX2* and *PGX3* are all widely expressed in expanding tissues, including seedlings, roots, leaves, and flower, and all of the loss-of-function mutant plants have growth defects (Rui et al. 2017; Xiao et al. 2017; Xiao et al. 2014). In *ltn-212*, mutation of *OsPG1* caused dark brown necrosis at leaf tips accompanied by decreased plant height, reduced spikelet length, and lower grain size and thousand seed weight relative to WT. This finding suggests that *OsPG1* is distributed in multiple tissues at different developmental stages. Given the necrotic phenotype of leaf tips, and changes of cell morphology in internodes and agronomic traits, we speculate that *OsPG1* affects apical development in rice. PGs comprise a large family, with 45 annotated PG-encoding genes in rice (Kim et al. 2006; Mccarthy et al. 2014). Phylogenetic analyses of PGs in rice revealed that *OsPG1* is classified in Clade A, genes in this clade are expressed in the main fruit or abscission zone (Hadfield and Bennett. 1998). This result is consistent with the GUS staining of grains. Therefore, we conclude that the Clade A genes contributed multiple functions and might thus play a unique role in cell wall remodeling in leaves and other tissues.

Plant cell walls are complex and composed of polysaccharides and protein polymers (Somerville et al. 2004), which are essential in monitoring plant-pathogen interactions (Cantu et al. 2008; Malinovsky et al. 2014; Bacete et al. 2018). Plant cell walls are the first obstacle for pathogens attempting to infect plant tissues and thus function as a passive defensive barrier. Recent studies have shown that plant cell walls have mechanisms for maintaining CWI, including diverse sensors and pattern recognition receptors (PRRs) in the plasma membrane. Alteration of CWI by impairment of proteins involved in biosynthesis or remodeling cell walls significantly affect disease resistance or abiotic stresses (Bellincampi et al. 2014; Miedes et al. 2014; Kesten et al. 2017). PGs, a type of endogenous pectinases, catalyze the degradation of pectin via cleaving HG backbones in plant cell walls (Peaucelle et al. 2012). PGs are normally defined as pathogenicity factors, which are secreted by pathogens to facilitate degradation of the cell wall barrier and promote pathogenesis. However, plants also produce PG. In *Arabidopsis*, knockdown and overexpression of *ADPG2* impacts resistance to *P. syringae* (Wang et al. 2017). In *ltn-212*, we found that incorrect splicing of *OsPG1* enhances the resistance to bacterial blight pathogen, meaning that the CWI could be altered in *ltn-212* plants. The cell wall remodeling also results in PR gene expression. Ectopic activation of endogenous pectin degrading enzyme ADPG1 induces release of pectic oligosaccharide elicitors of PR gene expression (Gallegogiraldo et al. 2020). Similarly, we also detected that some PR genes were upregulated or down-regulated, which may be associated with CWI alteration. Our results highlight the importance of CWI in plant-pathogen interactions.

Pectins are a complex family of polysaccharides that make up a significant fraction of the plant primary cell wall and middle lamella (Bonnin et al. 2014; Atmodjo et al. 2013). Previous studies have shown that pectin has important functions involved in pollen tube growth, fruit softening, providing structural support, and promoting cell-to-cell adhesion and defense responses (Marinrodriguez et al. 2002; Wolf et al. 2009; Hongo et al. 2012). Many examples of disease susceptibility/resistance phenotypes have been reported. Mutations in pectate lyase-like gene *PMR6* alter the composition of the plant cell wall and enhance resistance to powdery mildew in *Arabidopsis* (Vogel et al. 2002). Screening of mutants with alterations in cell wall monosaccharides indicated an important function of pectic polymers for penetration resistance and hyphal growth of *Colletotrichum higginsianum* at the biotrophic phase (Engelsdorf et al. 2016). Three pectin methylesterase genes *AtPMEI10*, *AtPMEI11* and *AtPMEI12* function as mediators maintaining CWI in *Arabidopsis* immunity (Lionetti et al. 2017). In this study, we observed that the thicknesses of the primary and secondary cell walls were significantly reduced in *ltn-212* compared with WT. The changes in cell walls were probably caused by the loss of OsPG1 function, which leads to the degradation of pectin. Consistent with this, the cell numbers increased almost threefold, while the cell size and shape significantly decreased. These results indicate that the function of *OsPG1* affects not only the cell wall, but also cell morphology.

Taking these findings together, it is clear that *OsPG1* regulated cell death and plant immunity systems through cell wall remodeling. But given the complexity of cell walls, the details in CWI-mediated immunity are still incompletely understood.

## Conclusion

*OsPG1* functions as a suppressor of programmed cell death and affects CWI, which regulates plant immunity. This finding facilitates efforts to understand the biological functions of PGs, and the role of the cell wall in mediating defense responses.

## Methods

### Plant Materials and Growth Conditions

The wild-type used in this study was ‘JiaHe212’(*Oryza sativa* L. *ssp. japonica* cv). The *ltn-212* mutant was obtained from the mutant population generated by EMS treatment of JiaHe212. All plants used in this study were grown in paddy under natural conditions in Hangzhou, Zhejiang province and Lingshui, Hainan province, China. For disease characterizations, the plants were grown in a greenhouse with a photoperiod of 12h white light at 30 °C and 12 h dark at 25 °C.

### Construct for Complementation and CRISPR Vectors and Rice Transformation

For the genetic complementation test, a 5790-bp genomic fragment covering the entire coding sequence and 2826-bp native promoter and 1098-bp terminator regions was amplified from the Japonica rice variety Jiahe212 genomic DNA by using KOD FX (Toyobo). The full length product was inserted to the pCAMBIA1300 vector using an In-Fusion Advantage Cloning kit (catalog no. PT4065; Clontech). For *OsPG1* knock-out vector construct and rice transformation entrusted BioRun company. The primer sequences used in this experiment listed in Additional file 1: Table S1.

### RNA Extraction and Quantitative Real-Time PCR (qRT-PCR) Analysis

Total RNA was extracted from rice leaves, roots, stems at different developmental stages using the RNAprep Pure kit for plants (Tiangen, China). Frist-strand cDNA was synthesized with a ReverTra Ace qPCR RT kit (Toyobo). The qRT-PCR assays were performed with Lightcycler 480 SYBR green (Roche, Sweden) using a LightCycler 480 II real-time PCR instrument (Roche, Sweden). The expression level of the target genes was normalized to that of the UBQ gene. The sequences of all primers used for qRT-PCR are given in Additional file 1: Table S1.

### GUS Assay

A 2.8-Kb native promoter region of *OsPG1* was amplified and inserted to the pCAMBIA 1305 vector. The transgene rice tissues were stained with GUS staining solution (50 mM PBS buffer; 10 mM EDTA, pH 8.0; 0.1% Triton X-100; 1 mg/mL X-gluc; 1 mM potassium ferricyanide; 1 mM potassium ferrocyanide). After incubating at 37 °C in the dark for 12 h, the chlorophyll was removed by boiling in 95% ethanol until removed completely.

### DAB and Evans Blue Staining and Measurement of Enzymatic Activity Related Parameters

Top leaves were collected at heading stage and immediately submerged in 10% SDS. After 10 min, the leaves were subsequently submerged in a 1 mg/mL solution of DAB or 0.5 mg/mL solution of Evans Blue incubated for 8 h in the dark at room temperature, respectively. Samples were then decolored in 95% boiling ethanol for 30 min and soaked for 48 h in 95% ethanol until all of the chlorophyll had been removed. The cleared leaves were then photographed.

Malondialdehyde (MDA) content, Peroxidase (POD), Super Oxide Dismutase (SOD), Catalase (CAT) activity, Soluble Protein (SP) and H_2_0_2_ content of wild type and *ltn-212* top leaves were measured from heading stage plants and using kits from Nanjing Jiancheng Bioengineering Research Institute.

### Transmission Electron Microscopy (TEM) and Cell Wall Composition Analysis

The materials were cut into small pieces and fixed in 2.5% glutaraldehyde and 0.1 M phosphate buffer at 4 °C overnight and washed three times in the phosphate buffer (0.1 M, pH 7.0) for 15 min at each step; postfixed with 1% OsO_4_ in phosphate buffer for 1-2 h and washed three times in the phosphate buffer (0.1 M, pH 7.0) for 15 min at each step. For TEM, the samples were subsequently dehydrated through a graded series of ethanol and then embedded in acrylic resin. Ultrathin sections (70-90 nm) were double stained with uranyl acetate and lead citrate aqueous solutions and observed with a Hitachi H-7650 transmission electron microscope (JEOL) at 80 kV. The content assay of cell wall components was entrusted to Convinced-Test company (Nanjing, China).

### Sequences Alignment and Phylogenetic Analyses

The gDNA and cDNA sequences of *OsPG1* and *ospg1* were amplified from WT and *ltn-212* using KOD FX, respectively. All the sequence alignments were used MegAlign and Gendoc software. The amino acid sequences of the *OsPG1* and Homologous proteins were downloaded from NCBI BLAST server (http://www.ncbi.nlm.nih.gov/). Sequence alignment was performed with ClustalW (Additional file 1: Table S1). A neighbor-joining method implemented in MEGA5 was used to generate the phylogenetic tree; the bootstrap values indicated at the nodes in the phylogenetic tree are based on 1000 replications.

### Pathogen Infection

Rice plants were inoculated with *Xoo* by the leaf-clipping method. CR1, CR4, and PXO96 strains were separately suspended in distilled water and adjusted to 10^9^ viable cells/mL (OD 600 = 1). The Pathogen infection were performed as described previously (Liu et al. 2017).

## Supplementary Information

**Additional file 1: Figure S1.** Leaf tip necrosis of *ltn-212* at tillering stage. A. Phenotype of WT and *ltn-212* at tillering stage (scale bar = 10 cm). B. Leaf tip necrosis identification of WT and *ltn-212* at tillering stage (scale bar = 5 cm). **Figure S2**. Data statistics of agronomic traits. A-B. Comparison of tiller numbers and plant height of WT and *ltn-212* (*n* = 10). C-D. Comparison of grain length and thousand grain weight of WT and *ltn-212* (n = 10). E. Comparison of panicles and culm length of WT and *ltn-212* (n = 10). ** indicates significance at *P* ≤ 0.01 and * indicates significance at *P* ≤ 0.05 (Student’s *t* test). **Figure S3**. Genome DNA and cDNA alignments between WT and *ltn-212*. Red star indicates start codon and stop codon, respectively. The red box indicates the incorrected splicing sequences. The red arrow indicates the mutant site. **Figure S4.** Amino acid sequence alignment of PG homologous. *ADPG2*: *Arabidopsis thaliana*, *BCMF9*: *Brassicarapa* L. ssp. *Pekinensis*, *CsPG1*: *Ciboria shiraiana*, *RSPG1*: *Rhizoctonia solani*, *SDPG*: *Glycinemax*. **Figure S5**. Phylogenetic analysis of *OsPG1* with other homologues in rice. **Figure S6**. Cell wall structure of leaf bottom, middle, and tip Statistical analysis of the primary cell wall, secondary cell wall, and middle lamella thicknesses of bundle sheath fiber cells in leave bottom(A), middle(B), and tip(C) of WT and *ltn-212*. Data are means ± SD of 20 cells. ** indicates significance at *P* ≤ 0.01. **Table S1.** Primers used in this study.

## Data Availability

All relevant data are provided within the article and its supplementary information files.

## Abbreviations

PG: Polygalacturonase
CWI: Cell wall integrity
ROS: Reactive oxygen species
PR: Pathogenesis-related
PCD: Programmed cell death
OGs: Oligogalacturonides
PME: Pectin methyl esterases
DAB: 3,3-Diaminobenzidine
MDA: Malonaldehyde
CAT: Catalase
POD: Peroxidase
SOD: Superoxide dismutase
*Xoo*: *Xanthomonas oryzae* pv. *oryzae*
WT: Wild type
ORF: Open reading frame
CDS: Coding sequence
GUS: β-glucuronidase
TEM: Transmission electron microscope
PRR: Pattern recognition receptor
