# Supplementary material for: OsPG1 Encodes a Polygalacturonase that Determines Cell Wall Architecture and Affects Resistance to Bacterial Blight Pathogen in Rice
Source: Rice (N Y). 2021 Apr 21;14:36. doi: 10.1186/s12284-021-00478-9 (PMC8060378; doi:10.1186/s12284-021-00478-9)
Supplement: Supplementary file 1 — Additional file 1: Figure S1. Leaf tip necrosis of ltn-212 at tillering stage. A. Phenotype of WT and ltn-212 at tillering stage (scale bar = 10 cm). B. Leaf tip necrosis identification of WT and ltn-212 at tillering stage (scale bar = 5 cm). Figure S2. Data statistics of agronomic traits. A-B. Comparison of tiller numbers and plant height of WT and ltn-212 (n = 10). C-D. Comparison of grain length and thousand grain weight of WT and ltn-212 (n = 10). E. Comparison of panicles and culm length of WT and ltn-212 (n = 10). ** indicates significance at P ≤ 0.01 and * indicates significance at P ≤ 0.05 (Student’s t test). Figure S3. Genome DNA and cDNA alignments between WT and ltn-212. Red star indicates start codon and stop codon, respectively. The red box indicates the incorrected splicing sequences. The red arrow indicates the mutant site. Figure S4. Amino acid sequence alignment of PG homologous. ADPG2: Arabidopsis thaliana, BCMF9: Brassicarapa L. ssp. Pekinensis, CsPG1: Ciboria shiraiana, RSPG1: Rhizoctonia solani, SDPG: Glycinemax. Figure S5. Phylogenetic analysis of OsPG1 with other homologues in rice. Figure S6. Cell wall structure of leaf bottom, middle, and tip Statistical analysis of the primary cell wall, secondary cell wall, and middle lamella thicknesses of bundle sheath fiber cells in leave bottom(A), middle(B), and tip(C) of WT and ltn-212. Data are means ± SD of 20 cells. ** indicates significance at P ≤ 0.01. Table S1. Primers used in this study. [file 12284_2021_478_MOESM1_ESM.docx]

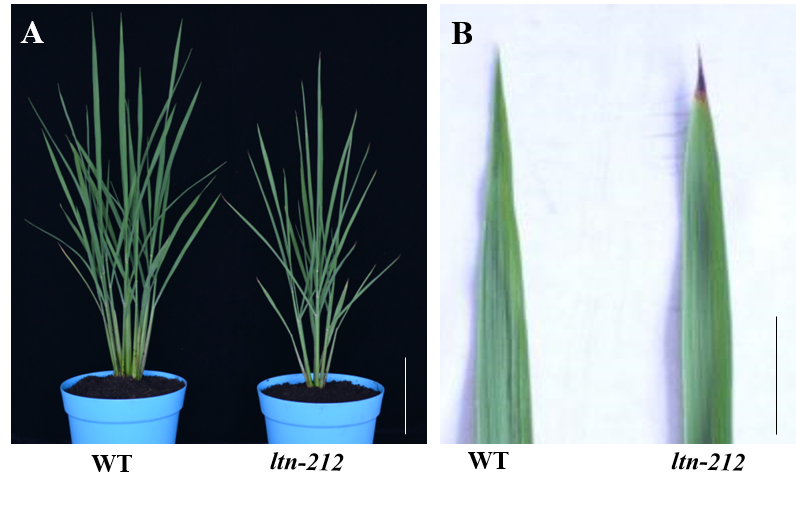
**Additional file 1: Figure. S1 Leaf tip necrosis of *ltn-212* at tillering stage**


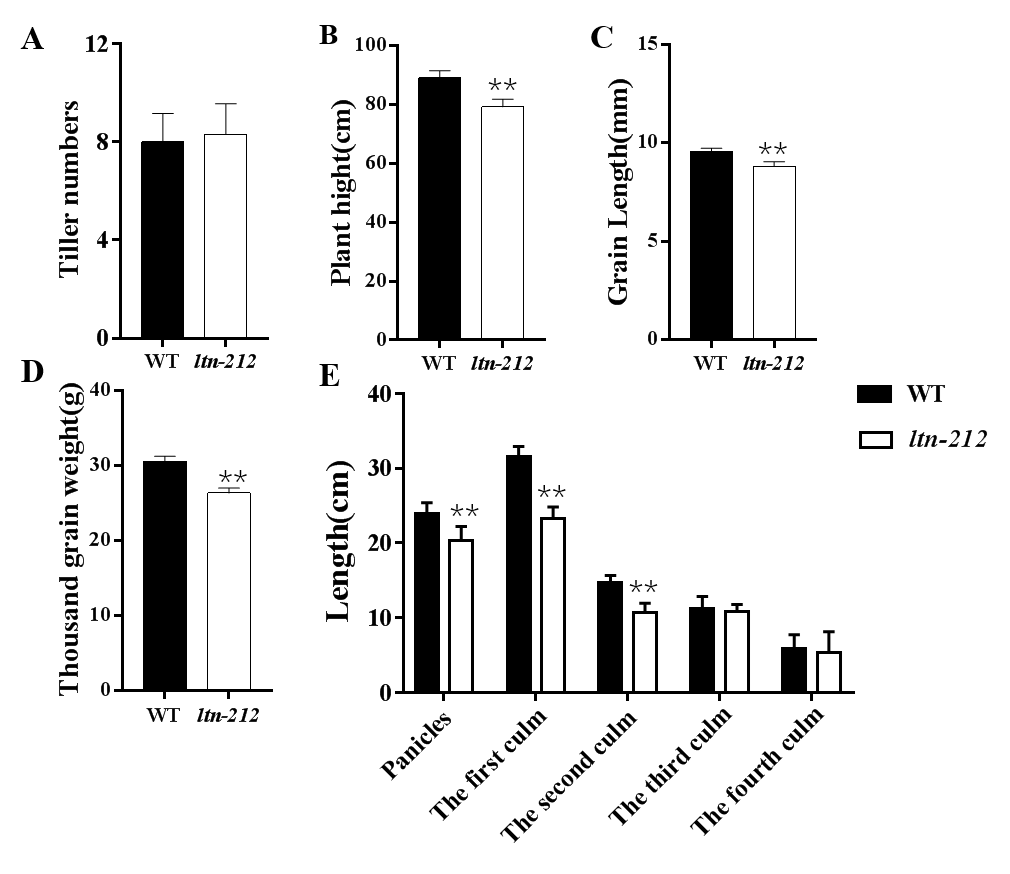


**Additional file 1: Figure. S2 Data statistics of agronomic traits**


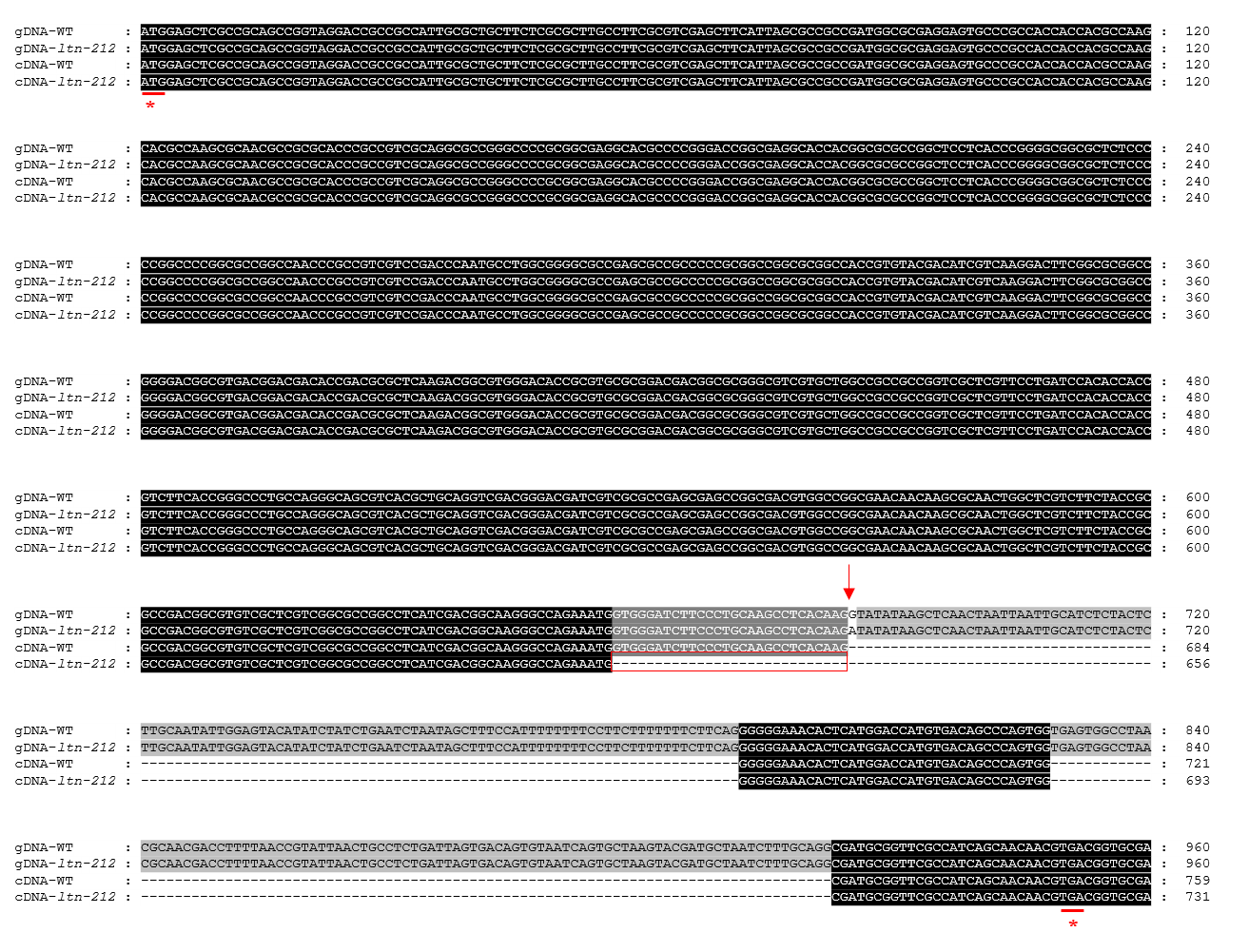
**Additional file 1: Figure. S3 Genome DNA and cDNA alignments between WT and *ltn-212***


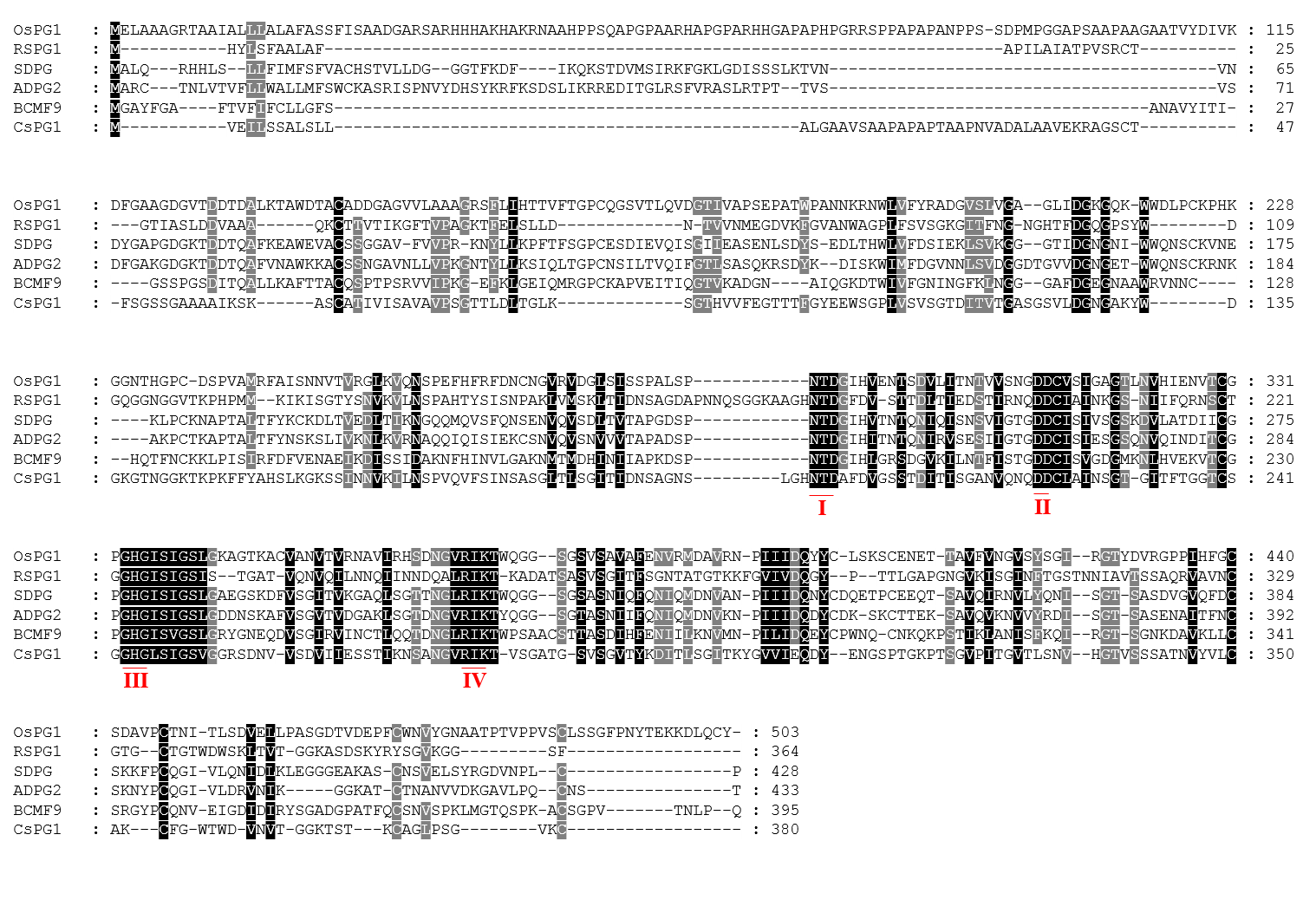
**Additional file 1: Figure. S4 Amino acid sequence alignment of PG homologous**


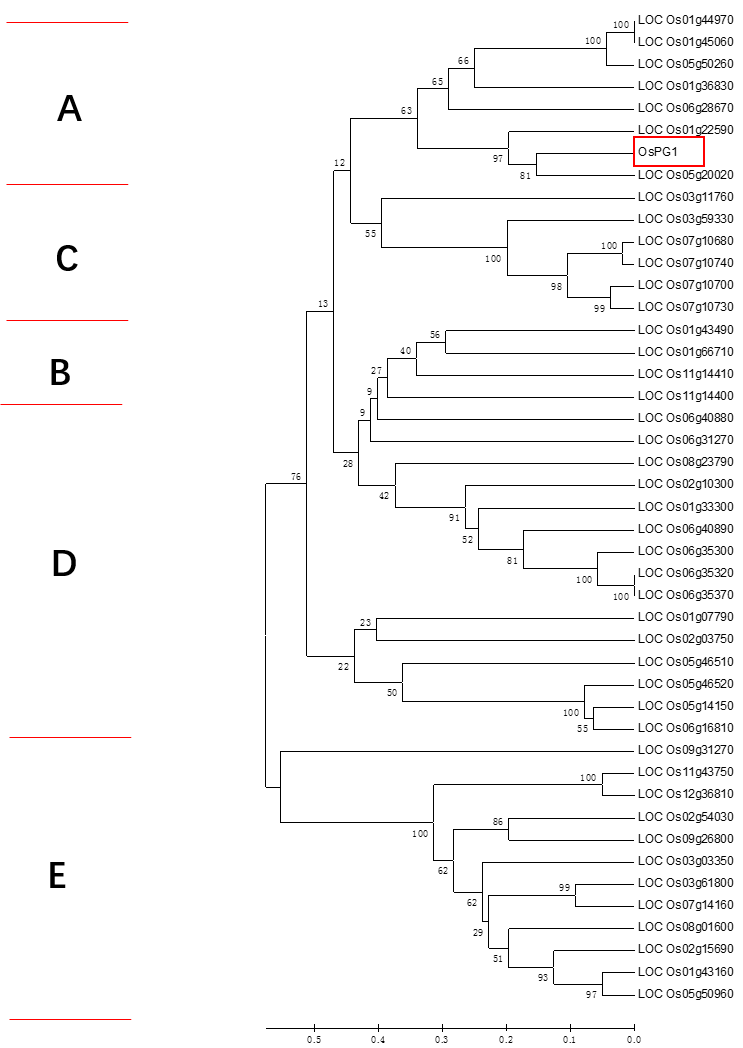


**Additional file 1: Figure. S5 Phylogenetic analysis of *OsPG1* with other homologues in rice**

**
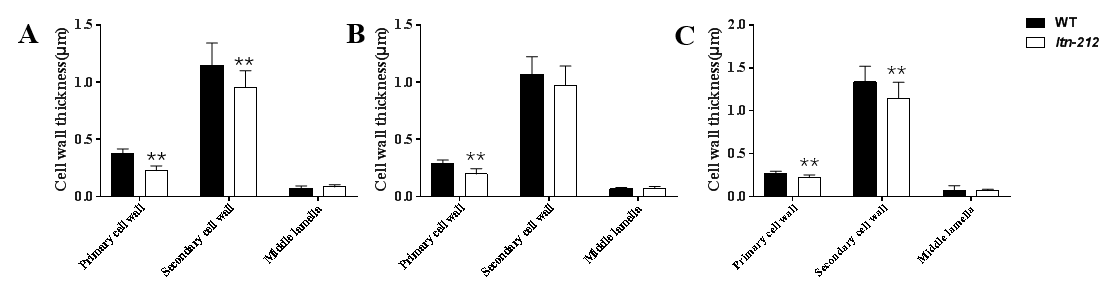
 Additional file 1: Figure. S6 Cell wall structure of leaf bottom, middle, and tip.**

**Additional file 1: Table S1. Primers used in this study**

| **Makers** | **Forward primer** | **Reverse primer** |
| --- | --- | --- |
| 1-12 | AAATGGGCTTCCTCCTCTTC | CAGCCTTGATCGGAAGTAGC |
| RM493 | GTACGTAAACGCGGAAGGTGACG | CGACGTACGAGATGCCGATCC |
| YSSR54 | CTATGCCTCCGCCTCTATCTCC | CTCTCTGGACCTACCCGTCATCC |
| YSSR41 | TTTCCCTGGTTTCAAGCTTACG | AGTACGGTACCTTGATGGTAGAAAGG |
| YSSR28 | ATGACTCTTCGCCATTCCATAGC | ACCATGGTCAGCCATCACTAGG |
| YJK15 | ATCCATGACCTGATCCTGAATCC | GACTTGACCAAACCCACAAGTGC |
| YJK26 | ATCCACCACAAACGACATTA | CGTGCAATCGCATTACACTA |
| YJK28  qPOX  qAOS2  qNPR1  qPR10 | GAGTGAATCTACGTTGGCTCAT  GCTCCAAGGTGAACTCCTAATT  AAGCTGCTGCAATACGTGTACTGG  CACTGCACTACGCCGTCGAAC  CTCAAGATGATCGAGGACTACC | ATCCTGCTAAAAGTTTGACAATA  ATATGGGTATATGTGGTGTGGC  CGACGAGCAACAGCCTTCCG  TCTCTTCGCCTCGCAGCAA  AGAAAGGCACATAAACACAACC |
| qJAZ1 | GCGCTCCCGGAGATGCCGAT | TTCGCTCGTTGTCGTGATCCTGT |
| qPR1a | CGTGTCGGCGTGGGTGT | GGCGAGTAGTTGCAGGTGATG |
| qPR1b | CATTGCTTTGGCCATGGTAG | GAACCCCAGAAGAGGTTCTC |
| qPR5 | CTTCTGCCCATAATGCATCATC | TGATTATCGATCAAGGTGTCGT |
| qPO-C1 | ATAGCAATGTGTACGTGGAGAT | ATTCCATGCACATACAGATGGA |
| UBQ | GCTCCGTGGCGGTATCAT | CGGCAGTTGACAGCCCTAG |
| *pOsPG1* | CCATGATTACGAATTCAGATGGATAAGGGCGTAAGG | GGCCAGTGCCAAGCTTCCCAA  CTGTTACTGGCTATGAC |
| *OsPG1*-pro | CGGTACCCGGGGATCCAGATGGATAAGGGCGTAAGG | CTCAGATCTACCATGGCACCGC  AGAAACCTATGAAA |
